# Frequency of Mucositis Among Head and Neck Cancer Patients Receiving Three-Dimensional (3D) Radiotherapy

**DOI:** 10.7759/cureus.9089

**Published:** 2020-07-09

**Authors:** Talha Laique, Ambreen Shabbir, Muhammad Ahmad Bilal Ahmadani, Maher Sohail Yaseen, Ayesha Ahmad, Jahanzeb Malik

**Affiliations:** 1 Pharmacology, Lahore Medical and Dental College, Lahore, PAK; 2 Pathology, Combined Military Hospital (CMH) Institute of Medical Sciences, Multan, PAK; 3 Radiology, Cardiac Centre, Bahawalpur, PAK; 4 Physiology, D.G Khan Medical College, Dera Ghazi Khan, PAK; 5 Radiation Oncology, Institute of Nuclear Medicine and Oncology (INMOL), Lahore, PAK; 6 Cardiology, Rawalpindi Institute of Cardiology, Rawalpindi, PAK

**Keywords:** 3-d radiotherapy, mucositis

## Abstract

Background and objective

Head and neck cancer can arise from any site like hypo-pharynx, oro-pharynx, lip, oral cavity, or larynx. Almost 90% of them are head and neck squamous cell carcinomas (HNSCC). In the current project, the goal was to determine the frequency of acute side effects in terms of mucositis during and immediately post-irradiation period in patients receiving concurrent three-dimensional (3D) radiotherapy in a tertiary care hospital.

Methodology

This descriptive case series with 106 enrolled patients was carried out from December 2019 to May 2020 at the Department of Radiation Oncology following approval. All patients were given radiotherapy or chemo-radio-therapy as per the clinician's advice and hospital protocol. All patients were evaluated at pre radiation time, at weekly intervals during treatment and at 11 weeks from 1st radiation fraction. Data was entered and analyzed by Statistical Package for Social Sciences, version 20 (SPSS Inc., Chicago, IL). Chi-square and Fisher's exact test was applied as p-value ≤ 0.05 was considered significant.

Results

In the present study, all patients (n=106) showed a mean age of 57.8 ± 8.3 years. There was a gradual increase in grades of mucositis in all patients after treatments until seven weeks. After one month of post-treatment, a decrease in grades of mucositis was observed in all patients.

Conclusion

Acute side effects appeared in all patients receiving 3D radiation therapy (RT) although the treatment response was good. Hence, we concluded it has a high incidence of treatment-related toxicities but it is safe.

## Introduction

Head and neck cancer is a heterogeneous disease that can arise from any site like hypo-pharynx, oro-pharynx, lip, oral cavity, nasopharynx, or larynx [1]. Almost 90% of them are head and neck squamous cell carcinomas (HNSCC). It is ranked sixth among all cancers globally with reported new patients (630,000) identified per year. Globally, 12% of all malignancies are constituted by oral and head and neck cancer (OHNC). Oral (400,000) and laryngeal (160,000) cancer cases usually develop each year according to previous estimation [2]. Head and neck cancers account for approximately 45%-55% survival rate for patients having it after the five years of diagnosis and treatment [3]. Males (21%) suffer more from head and neck cancers than females (11%) across the Pakistani population respectively [4,5].

Head and neck cancers are mainly due to various factors including demographic parameters (smoking, alcohol intake), eating patterns, infections with viruses, and family history [6]. Concurrent radiation therapy (RT) with cisplatin has improved locoregional control in squamous cell cancer. It is now the basic component of a multi-disciplinary approach for head and neck cancer treatment. The purpose of RT is to damage tumor maximally with minimum side effects using modalities like two-dimensional (2D)/three-dimensional (3D)/intensity-modulated radiation therapy (IMRT) though it is linked with several short-term and long-term side effects [7-9]. 

An adverse event (AE) is any unwanted effect produced as a result of a medical treatment or procedure. Toxicity criteria of the Radiation Therapy Oncology Group (RTOG) and the European Organization for Research and Treatment of Cancer (EORTC) defined acute side effects like mucositis, dermatitis, and dry mouth, etc into grades to show organ toxicity among subjects receiving RT [7].

Regarding the high incidence of head and neck cancers among our population with limited data available regarding acute side effects of RT, we planned the current study to see the frequency of acute side effects (mucositis) associated with 3D-radiotherapy technique (3D-RT) in head and neck cancers treatment. It helped us to observe how frequently mucositis developed among patients on RT for continuous 11 weeks. 

## Materials and methods

This descriptive case series with 106 enrolled patients was carried out from December 2019 to May 2020 at the Department of Radiation Oncology, Institute of Nuclear Medicine and Oncology (INMOL), Lahore, following the approval by the Hospital’s Ethical Committee. The Sample size (106) cases were calculated with a 95% confidence level, 9% margin of error, and taking an expected percentage of mucositis with 3D-RT as 33.3% (9). Patients were enrolled by non-probability consecutive sampling. All patients of head and neck cancer including both genders with a mean age of 57.8 ± 8.3 years presenting to the radio-oncology department were enrolled. Exclusion criteria involved patients who were unable to give informed consent, pre-existing skin diseases, any second malignancy or metastasis, and pregnant. Written informed consent was taken from the patient at the time of enrollment. 

Data was entered and analyzed by Statistical Package for Social Sciences, version 20 (SPSS Inc., Chicago, IL). Quantitative data like age (in years) and total radiation dose were presented as Mean± SD. The categorical data like gender, site of cancer, and acute side effects were offered as frequency and percentages. Chi-square and Fisher's exact test was used to compare the frequency of acute side effects among different based on gender and the site of cancer with p-value ≤ 0.05 was considered significant.

## Results

Among 106 enrolled patients, age ranged from 25-70 years. Demographic parameters like age and dose of radiation as mean± SD are shown in Table 1 below. All these parameters were noted at the time of enrollment.

**Table 1:** Descriptive statistics of patients with respect to age and total radiation dose

	Mean ± S.D	Minimum	Maximum
Age (years)	57.8 ± 8.3	25	70
Total radiation dose (Grays)	62.8 ± 7.4	30	70

The distribution of patients enrolled in the study concerning gender and sites of cancer involved is shown in Table 2 below.

**Table 2:** Distribution of participants with respect to gender and sites of cancer

Table 2: Distribution of participants with respect to gender and site of cancer			
Variable	Category	Frequency	Percentage (%)
Gender	Male	44	41.5
	Female	62	58.5
Site	Pharynx	23	21.7
	Hypopharynx	22	20.8
	Larynx	8	7.5
	Nasopharynx	22	20.8
	Oral cavity	31	29.2

There was a gradual increase in grades of mucositis in all patients after treatments with 3D-RT until seven weeks. After one month of post-treatment, a decrease in grades of mucositis in all patients was observed. The result regarding distribution according to grades of mucositis among enrolled patients are summarized in Table 3.

**Table 3:** Showing distribution of patients according to the grade of mucositis

Week	Grade 0	Grade 1	Grade 2	Grade 3	Grade 4	Grade 5
^1st^	79 (74.5%)	27 (25.5%)	0 (0%)	0 (0%)	0 (0%)	0 (0%)
2^nd^	64(60.4%)	42 (39.6%)	0 (0%)	0 (0%)	0 (0%)	0 (0%)
3^rd^	1 (0.9%)	79 (74.5%)	26(24.5%)	0 (0%)	0 (0%)	0 (0%)
4^th^	0 (0%)	78(73.6%)	28(26.4)	0 (0%)	0 (0%)	0 (0%)
5^th^	0 (0%)	38(35.8%)	67(63.2%)	1 (0.9%)	0 (0%)	0 (0%)
6^th^	0 (0%)	0 (0%)	79 (74.5%)	27 (25.5%)	0 (0%)	0 (0%)
7^th^	0 (0%)	0 (0%)	52 (49.1%)	49 (46.2%)	5 (4.7%)	0 (0%)
11^th^	56 (52.8%)	49 (46.2%)	1 (0.9%)	0 (0%)	0 (0%)	0 (0%)

Chi-square and Fisher's exact test was used to compare the frequency of mucositis grades between male and female patients (Table 4). The grades of mucositis in male patients versus female patients was significantly different on the 4th week of treatment alone. It means that mucositis of varying grades developed among patients at the 4th week of treatment more than any other week till the 7th week of treatment.

**Table 4:** Showing comparison of the grade of mucositis between both genders

Table 4: Showing comparison of grade of Mucositis between both genders								
Week	Gender	Grade 0	Grade 1	Grade 2	Grade 3	Grade 4	Grade 5	p-value
1^st^	Male	29 (65.9%)	15 (34.1%)	0 (0%)	0 (0%)	0 (0%)	0 (0%)	0.086
	Female	50 (80.6%)	12 (19.4%)	0 (0%)	0 (0%)	0 (0%)	0 (0%)	
4^th^	Male	0 (0%)	28(63.6%)	16 (36.4%)	0 (0%)	0 (0%)	0 (0%)	0.050*
	Female	0 (0%)	50(80.6%)	12(19.4%)	0 (0%)	0 (0%)	0 (0%)	
7^th^	Male	0 (0%)	0 (0%)	22(50%)	20(45.5%)	2(4.5%)	0 (0%)	>0.999
	Female	0 (0%)	0 (0%)	30(48.4%)	29(46.8%)	3(4.8%)	0 (0%)	
11^th^	Male	0 (0%)	23(52.3%)	21(47.4%)	0 (0%)	0 (0%)	0 (0%)	>0.999
	Female	0 (0%)	33(53.2%)	28(45.2%)	1(1.6%)	0 (0%)	0 (0%)	

Chi-square and Fisher's exact test was used to compare the frequency of mucositis grades among sites at 1st, 4th and 7th week of treatment as shown in Table 5. This was done in order to observe the development of mucositis at start after the 1st week of treatment and at end of treatment at the 7th week involving various sites in head and neck region. In the current study, we observed it one month post treatment at the 11th week to see improvement in its grades. There was a significant difference in the frequency of mucositis grades among sites involved in the 7th week of treatment. This showed grade 2 and 3 mucositis developed at all sites of head and neck region.

**Table 5:** Showing comparison of the grades of mucositis among sites involved

Table 5: Showing comparison of grades of Mucositis among sites involved								
Week	Site	Grade 0	Grade 1	Grade 2	Grade 3	Grade 4	Grade 5	p-value
1^st^	Pharynx	14 (60.9%)	9(39.1%)	0 (0%)	0 (0%)	0 (0%)	0 (0%)	0.321
	Hypopharnx	15 (68.2%)	7(31.8%)	0 (0%)	0 (0%)	0 (0%)	0 (0%)	
	Nasopharyx	17 (77.3%)	5(22.7%)	0 (0%)	0 (0%)	0 (0%)	0 (0%)	
	Oral cavity	26 (83.9%)	5(16.1%)	0 (0%)	0 (0%)	0 (0%)	0 (0%)	
7^th^	Pharynx	0 (0%)	0 (0%)	6 (26.1%)	14(60.9%)	3(13.0%)	0 (0%)	0.012^*^
	Hypopharnx	0 (0%)	0 (0%)	13 (59.1%)	8 (36.4%)	1(4.5%)	0 (0%)	
	Nasopharyx	0 (0%)	0 (0%)	12 (54. 5%)	10 (45.5%)	0 (0%)	0 (0%)	
	Oral cavity	0 (0%)	0 (0%)	20 (64.5%)	11 (35.5%)	0 (0%)	0 (0%)	
11^th^	Pharynx	0 (0%)	7(30.4%)	15 (65.2%)	1 (4.3%)	0 (0%)	0 (0%)	0.104
	Hypopharynx	0 (0%)	13(59.1%)	9 (40.9%)	0 (0%)	0 (0%)	0 (0%)	
	Nasopharyx	0 (0%)	12(54.5%)	10 (45.5%)	0 (0%)	0 (0%)	0 (0%)	
	Oral cavity	0 (0%)	21(67.7%)	10 (32.3%)	0 (0%)	0 (0%)	0 (0%)	

## Discussion

RT was planned for patients with HNSCC. It was done to determine the frequency of acute side effects in terms of mucositis during and immediately post-irradiation period (upto 11 weeks from the first fraction) in patients receiving concurrent 3D-RT. Thus we examined the efficacy and safety of RT in non-metastatic, stage I to stage III HNSCC patients, very representative of our daily practice in our country Pakistan [10]. There was a significant clinical response to RT in our patients. No death was reported in our groups. Acute side effects related to RT are not reviewed routinely in our setups. Pakistan is one of the high burden countries for head and neck cancers. 

Our enrolled sample size was 106 in the current project. The number of patients conformed with one another previous study who in their project enrolled 221 patients to determine the presenting symptoms in head and neck cancer patients [11]. In contrast, one study carried on the Brazilian population included 30 head and neck cancer patients [10]. 

Age (mean ± SD) of enrolled patients in our study was 57.8 ± 8.3 years in conformity with the previous study where age (mean ± SD) of enrolled head and neck cancer patients among European population was 64 ± 12 years (Table 1). In past researches, the median age was 53 (37-68) rather than mean of age [12].

In the current study, tumors involving various head and neck sites like pharynx, hypopharynx, larynx, larynx, and oral cavity were considered. The most common site was oral cavity 31 (29.2%) and the least common site was larynx 8 (7.5%) as given in Table 2. Our results were in line with previous studies [10].

In our project, acute side effects like mucositis were seen during and immediately after post-treatment at four weeks interval. There was a gradual increase in grades of mucositis in all patients after treatments until seven weeks. Mucositis of grade 1 was observed in only 27 (25.5%) patients after one week of treatment. At the 4th week after treatment, mucositis of grade 1 and 2 was observed in 106 (100.0%) patients. At the 7th week of treatment, mucositis of grade 4 was observed in five (4.7%) patients, mucositis of grade 2 and 3 was observed in 52 (49.1%) and 49 (46.2%) patients, respectively. After one month of post-treatment, a decrease in grades of mucositis in all patients was observed as shown in Table 3. Our findings were in line with previous studies of head and neck cancer treatment with chemo-radiotherapy [13].

Our study had several limitations like financial constraints and fewer resources. We did not perform positron emission tomography (PET) scan and genetic study to see genetic variability among enrolled subjects.

## Conclusions

Acute side effects appeared in all patients receiving 3D-RT although the treatment response was good. Hence, we concluded 3D-RT has a high incidence of treatment-related toxicities but is relatively safe. Mucositis of varying grades developed till the completion of therapy but improved later at one-month post treatment. It was a reversible therapy-related toxicity but not life threatening.
